# [Retracted] Alpinetin suppresses proliferation of human hepatoma cells by the activation of MKK7 and elevates sensitization to cis‑diammined dichloridoplatium

**DOI:** 10.3892/or.2023.8534

**Published:** 2023-03-27

**Authors:** Bo Tang, Jian Du, Jingwen Wang, Guang Tan, Zhenming Gao, Zhongyu Wang, Liming Wang

Oncol Rep 27: 1090–1096, 2012; DOI: 10.3892/or.2011.1580

Following the publication of the above paper, it was drawn to the Editors' attention by a concerned reader that western blot data shown in Fig. 2B were strikingly similar to data that had appeared in different form in another article. Owing to the fact that the contentious data in the above article were already under consideration for publication elsewhere prior to its submission to *Oncology Reports*, the Editor has decided that this paper should be retracted from the Journal. The authors were asked for an explanation to account for these concerns, but the Editorial Office did not receive a reply. The Editor apologizes to the readership for any inconvenience caused.

